# A softening laminar electrode for recording single unit activity from the rat hippocampus

**DOI:** 10.1038/s41598-019-39835-6

**Published:** 2019-02-20

**Authors:** A. Zátonyi, G. Orbán, R. Modi, G. Márton, D. Meszéna, I. Ulbert, A. Pongrácz, M. Ecker, W. E. Voit, A. Joshi-Imre, Z. Fekete

**Affiliations:** 1 Research Group for Implantable Microsystems, Faculty of Information Technology & Bionics, Pázmány Péter Catholic University, Budapest, Hungary; 2 Doctoral School of Chemical Engineering and Material Sciences, University of Pannonia, Veszprém, Hungary; 3 Microsystems Laboratory, Institute for Technical Physics & Material Sciences, Centre for Energy Research, Hungarian Academy of Sciences, Budapest, Hungary; 4 Óbuda University Doctoral School on Materials Sciences & Technologies, Budapest, Hungary; 5 Institute of Cognitive Neuroscience and Psychology, Research Centre for Natural Sciences, Hungarian Academy of Sciences, Budapest, Hungary; 6 Advanced Polymer Research Laboratory, The University of Texas at Dallas, Richardson, TX USA

## Abstract

Softening neural implants that change their elastic modulus under physiological conditions are promising candidates to mitigate neuroinflammatory response due to the reduced mechanical mismatch between the artificial interface and the brain tissue. Intracortical neural probes have been used to demonstrate the viability of this material engineering approach. In our paper, we present a robust technology of softening neural microelectrode and demonstrate its recording performance in the hippocampus of rat subjects. The 5 mm long, single shank, multi-channel probes are composed of a custom thiol-ene/acrylate thermoset polymer substrate, and were micromachined by standard MEMS processes. A special packaging technique is also developed, which guarantees the stable functionality and longevity of the device, which were tested under *in vitro* conditions prior to animal studies. The 60 micron thick device was successfully implanted to 4.5 mm deep in the hippocampus without the aid of any insertion shuttle. Spike amplitudes of 84 µV peak-to-peak and signal-to-noise ratio of 6.24 were achieved in acute experiments. Our study demonstrates that softening neural probes may be used to investigate deep layers of the rat brain.

## Introduction

Implantable microelectrodes interface with neuronal populations by means of electrical, optical and chemical transduction^1^. Addressing long-term stability of these devices inside the brain tissue is of utmost importance to enhance the utility in clinical settings. A major reason for device failure is foreign body response, where encapsulation by astrocyte cells is associated with a reduced population of neurons in the vicinity of the brain-electrode interface. Various strategies have been explored to alleviate this neuroinflammatory response to the implantation of microelectrodes^2^. Injection of anti-inflammatory agents into the extracellular space or tuning the surface topography and chemistry have been proposed to reduce foreign body response. For example, microglia activation was shown to be reduced by the controlled administration of anti-inflammatory drugs^3^. In another work, the proliferation of glial cells responsible for the encapsulation and failure of these devices was mitigated by nanostructuring the surface of the implant^4^. Changing the chemical properties of the surface has been also proposed (e.g. introducing polymer coatings), however, the influence of these films on chronic performance of coated microelectrodes is debated^5^. Typical mechanical failures of microprobes may be induced either during insertion of the device into the neural tissue or in response to micromotions occurring around chronically implanted interfaces inside the skull. Penetrating the tissue can also provoke great neuronal loss due to interfacial forces dependent on device geometry and surgical conditions^6^ and contribute to the manifestation of the foreign body response in long term *in vivo* experiments^7^. Due to the recent advances in both material science and technology, the application of flexible, soft and stretchable materials has gained attention in the development of these microsystems^8^. Indeed, the mechanical interaction between the device and the surrounding cells may be a key factor in provoking the formation of glial sheath and in hindering longevity of recording or stimulation functions of brain implants. The physical mismatch between non-compliant implant substrates (e.g. tungsten, stainless steel or silicon) and the tissue induces permanent mechanical stress in the surrounding tissue. Replacing these conventional substrates with more adaptive or responsive materials can significantly reduce strains and the inherent micromotion induced stresses^9,10^. They also proved that glial encapsulation has an effect on the long-term changes in the mechanical mismatch^11^. Nguyen *et al*. have found histological evidence on the reduction of neuroinflammatory response to soft neural probes in comparison with stiff ones^12^. The same study also demonstrated that the soft implants at 12.7 MPa Young’s modulus, tethered in rat brain, have fully recovered from neuron loss in the immediate vicinity of the probes by sixteen weeks post-implantation. This suggests that tissue compliant probes can be fabricated from materials presenting elasticity in the order of 10 MPa and perhaps above. Materials and technologies for soft neuroprosthesis so far demonstrated good tolerance of the host tissue and a potential for long-term neurotherapies^13^.

Recently, several newly-engineered substrate materials have been proposed as mechanically adaptive components for soft neuroprostheses. These polymeric materials undergo chemical or temperature based activation resulting in a lower Young’s modulus after placed in the living tissue, but they maintain a Young’s modulus of several GPa and provide easy handling before and during implantation. Cellulose nanofibers embedded in a PVA-matrix showed remarkable changes in elastic properties between dry (4–5 GPa) and wet state (12 MPa)^14,15^. Thermoset shape memory polymers (SMP) that can be tuned to undergo glass transition upon implantation are presently being explored. A thermally reactive copolymer made of methyl acrylate (MA) and isobornyl-acrylate (IBoA) cross-linked with PEG diacrylate was reported as neural probe substrate capable of softening from a Young’s modulus of 700 MPa to 300 kPa^16^. Thiol-click chemistries have been proposed with the advantage of a widely tunable network structure and response to physiological conditions (change in temperature and fluid uptake)^17^. In particular, thiol-ene/acrylate substrate compositions have been shown to soften from over 1 GPa to 18 MPa with less than 3% fluid uptake upon exposure to 37 °C PBS; and cortical activity recording in rat subjects was demonstrated with probes built on such substrate^18^. Later, intracortical probes of thiol-ene/acrylate substrate that soften to a predicted 50 MPa were demonstrated to have neural recording capability over two months *in vivo*^19^. Leveraging the feasibility of softening polymers for recording and modulation of neural activity, several studies have shown that mechanically adaptive materials are also good candidates to act as substrate materials for multimodal microdevices used for optogenetic^20^ or pharmacologic intervention^21^.

In previous works, the Voit Lab has presented *in vitro* and *in vivo* biocompatibility test results assessing cytotoxicity and neurotoxicity of the substrate SMP material^19,22–24^. Cytotoxcity tests using NCTC fibroblasts and primary cortical neurons^24^, and neurotoxicity tests using MEA-based functional assays^19,24^ and *in vitro* glial scarring assays^24^ were carried out. The SMP materials were found to pass *in vitro* cytotoxicity assays, and neither single channel or network activity was found to be significantly altered. SMP-coatings were at least as well tolerated as bare stainless steel^24^. *In vivo* neuronal loss and astrocyte activation were found comparable to that previously reported for silicon probes^22,23^.

Most of the SMP brain probes in the literature comprised of a simple, three-layer device architecture of polymer, gold and Parylene-C, where the polymer layer was cast to thickness. Only a single study proposed a material configuration similar to ours^22^. In our work, a novel fabrication process flow is developed based on spin-coating, which allows precise control of the SMP thickness and the application of a top layer coating. The goal of this work was to (1) demonstrate intracortical probes where the softening polymer is presented on both sides of the probe, and (2) demonstrate implantation into deeper brain structures in rat. The presentation of the softening polymer on both sides of the penetrating probes provides uniform interfacing with the biological tissue. Implanting longer probes, 5 mm long in this work, is a step towards building human-sized interfaces. In order to minimize the footprint of the implants, insertion mechanics will need to be optimized by adjustments to the probe structure (laminar layer arrangements and thicknesses), which will be possible with this new microfabrication technology. Here, we report a robust microfabrication and packaging technology of penetrating neural probes, and demonstrate targeted implantation into the hippocampus of rat subjects. Material synthesis, fabrication details, electrochemical testing in saline solution and acute *in vivo* performance of the constructed probes are presented.

## Experimental

### Synthesis and characterization of the substrate material

Our softening polymer microelectrode probes were prepared with a thiol-ene/acrylate polymer composition reported earlier by the Voit group^19,20^ whereby the monomer solution consisted of a stoichiometric ratio of tri-functional thiol and tri-functional ene molecules, with an additional 31 mol % of bi-functional acrylate component. Namely, 1,3,5-triallyl-1,3,5-triazine-2,4,6(1 H,3 H,5 H)-trione (TATATO) and tricyclo[5.2.1.0]^2,6^ decanedimethanol (TCMDA) were purchased from Sigma-Aldrich, and Tris[2-mercaptopropionyloxy)ethyl]isocyanurate (TMICN) was purchased from Bruno Bock. 2,2-Dimethoxy-2-phenylacetophenone (DMPA) was purchased from Sigma-Aldrich and used as photoinitiator in 0.1 wt %. The monomer solution was prepared in stages as follows: first, the DMPA crystals were dissolved in TATATO by centrifugal mixing using a FlackTek DAC150 at 3000RPM for 15 min. TCMDA was added and mixed for 5 min in a second stage, and TMICN was added and mixed in a final, third stage. The monomer solution was then spin-coated on silicon wafers, and UV polymerized immediately in UVP CL-1000 model ultraviolet crosslinker ovens. Polymerization involved a short (30 seconds) illumination with 254 nm light followed by a long (60 minutes) illumination with 365 nm light. The films were post-cured in 120 °C vacuum oven for 24 hours.

Dynamic mechanical analyses (DMA) of thin film polymer samples was performed in tension, using an RSA-G2 Solids Analyzer by TA Instruments, as demonstrated earlier (Ecker 2017). Wet measurements employed an immersion bath filled with phosphate buffered saline (PBS) solution, where the samples were soaked for 1 hour at 37 °C before cooling them to 20 °C to start the measurement. For both dry and wet measurements, 30 µm thick and 4.5 mm wide polymer strips were clamped in 15 mm distance, tensed with a 0.2 N preload force, and measured with an oscillating strain amplitude of 0.275% at 1 Hz. Temperature ramping employed heating at 2 °C/min. Storage modulus and tan(delta) with respect to temperature were investigated. Soak testing, to show real time polymer softening, involved immersing a dry sample in PBS at room temperature (24 °C), heating to 37 °C in approximately 1500 seconds, and holding the temperature. All DMA samples were prepared using the hard masking and plasma etching processes described in the microfabrication section.

### Microfabrication

Length, width and thickness of the eight-channel laminar microelectrode was 5 mm, 200 µm and 60 µm, respectively. Recording sites had a diameter of 15 µm with a spacing of 188 microns. The first site was positioned at a distance of 150 microns from the probe tip.

**Figure 1:**
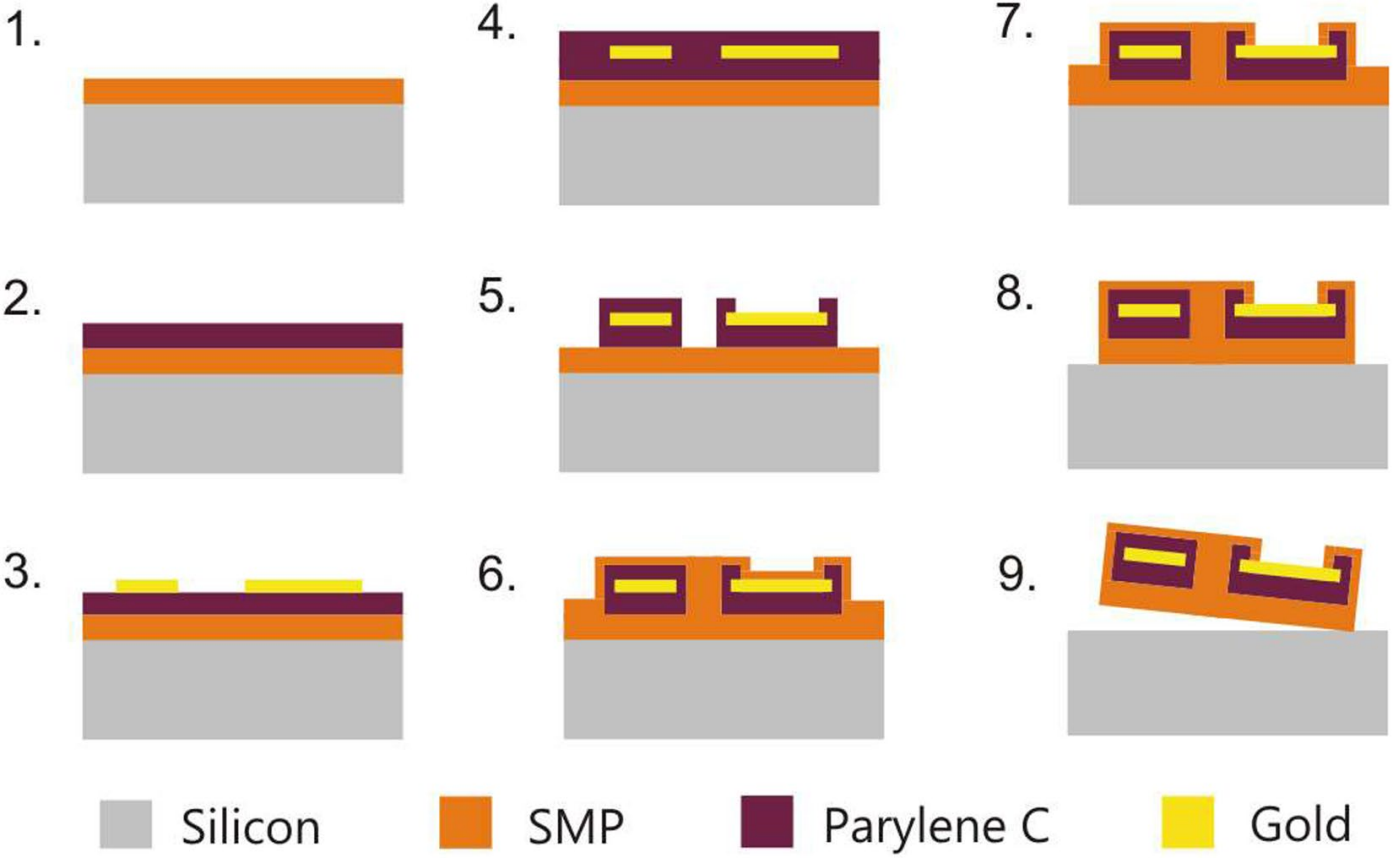
Schematic of the process flow of probe fabrication.

The softening polymer microelectrode probes were fabricated using standard MEMS processes provided by the clean room facility of The University of Texas at Dallas and that of the Hungarian Academy of Sciences. The process flow is shown on Fig. 1. The steps of fabrication were as follows. A first layer of 53 µm thick softening polymer film was spin-coated on a Si handle wafer and cured as described earlier (step 1 on Fig. 1). A 500 nm thick Parylene-C film was deposited on top at room temperature using the Gorham process in an SCS Labcoater 2 Parylene deposition system (step 2). This was followed by a gold lift-off process (step 3) using a bilayer of plasma enhanced chemical vapor deposited (PECVD) silicon nitride (deposited in a Plasma-Therm 790 at 150 °C substrate temperature) and S1813 photoresist, where fluorine plasma RIE was used for pattern transfer into the silicon nitride, creating approximately one micron undercut, and the gold was e-beam evaporated. Lift-off involved soaking in AZ-400K (MicroChemicals GmbH, Germany), followed by a diluted hydrofluoric acid (HF) soak to remove the nitride. This step provided 400 nm thick gold traces to become electrode sites, wiring, and bonding pads of the probes. Another 500 nm thick Parylene-C film was deposited (step 4), and patterned using reactive ion etching (RIE) in oxygen plasma masked with S1818 photoresist (step 5). The total 1 µm thick Parylene-C was etched in this step to maintain Parylene-C coverage in proximity to the gold patterns only (overlaying with 2 um offset). The wafer was then coated with 7 µm thick softening polymer on top (step 6). PECVD silicon nitride was deposited, patterned and used as a hard mask for two consecutive oxygen plasma etches. In both cases, a fresh layer of PECVD silicon nitride was deposited, and patterned with fluorine plasma RIE as defined by photolithographically patterned S1813 photoresist. In the first polymer etch, electrode sites and bonding pads were etched clean of the polymers using a capacitively coupled plasma process in a Technics Series 800 RIE at 200 mTorr and 200 W (step 7). Second, high density oxygen plasma was employed using an Oxford Plasmalab 100 deep reactive ion etching equipment in order to etch the contour of the probes (step 8). ICP and RF power were set to 1500 W and 30 W, respectively. Oxygen flow of 45 sccm was maintained in the vacuum chamber (15 mTorr) at 15 °C. During the etch process, the backside of the handle wafer was cooled by a permanent He flow (10 Torr). The masking layers were removed in acetone and buffered HF. The structures were released from the handle wafer underwater (step 9).

### Packaging

**Figure 2:**
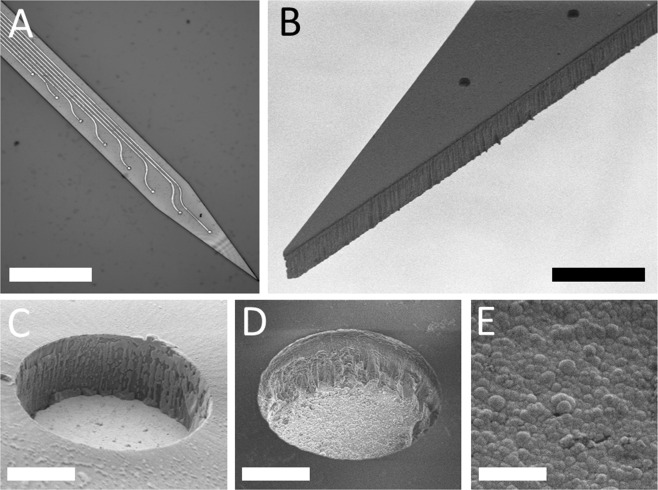
(**A**) Ready-to-use neural probe for hippocampus recording. Scale bar is 3 mm. (**B**) Schematic cross-sectional view of the layer structure used to connect bonding pads to pins of A79070 Omnetics connectors. Figure is not to scale.

To make a firm and leak-free connection between the bonding pads of the electrode and the external connector, a custom process flow was determined. The final composition of packaging materials is shown in Fig. 2. Drying or curing phase in all process step was performed at room temperature for at least 24 hours which is well below the transition temperature to avoid any thermal shock which would lead to the failure of the probe structure. First, a drop of CW2400 silver epoxy glue (Chemtronics, US) was dispensed onto the center surface of the gold bonding pad was allowed to dry. In order to separate the gold pads from the water-permeable substrate, Elastosil RT 607 (Wacker Chemie AG, Germany) was applied as a thin even layer. After 24 hours drying at room temperature, a second drop of silver epoxy glue was dispensed right above the first drop. The connector (A79040-001, Omnetics Connector Corp., MI, USA) pin was gently pressed into the second drop and was allowed to cure. In the last phase, all surfaces around the connector pins were preserved by Araldite 1401 adhesive (Huntsman Advanced Materials, TX, USA). Since the adhesion between Araldite and Elastosil is weaker than that between Araldite and the substrate, an extra amount of Araldite was deposited beyond Elastosil-covered surface.

**Figure 3:**
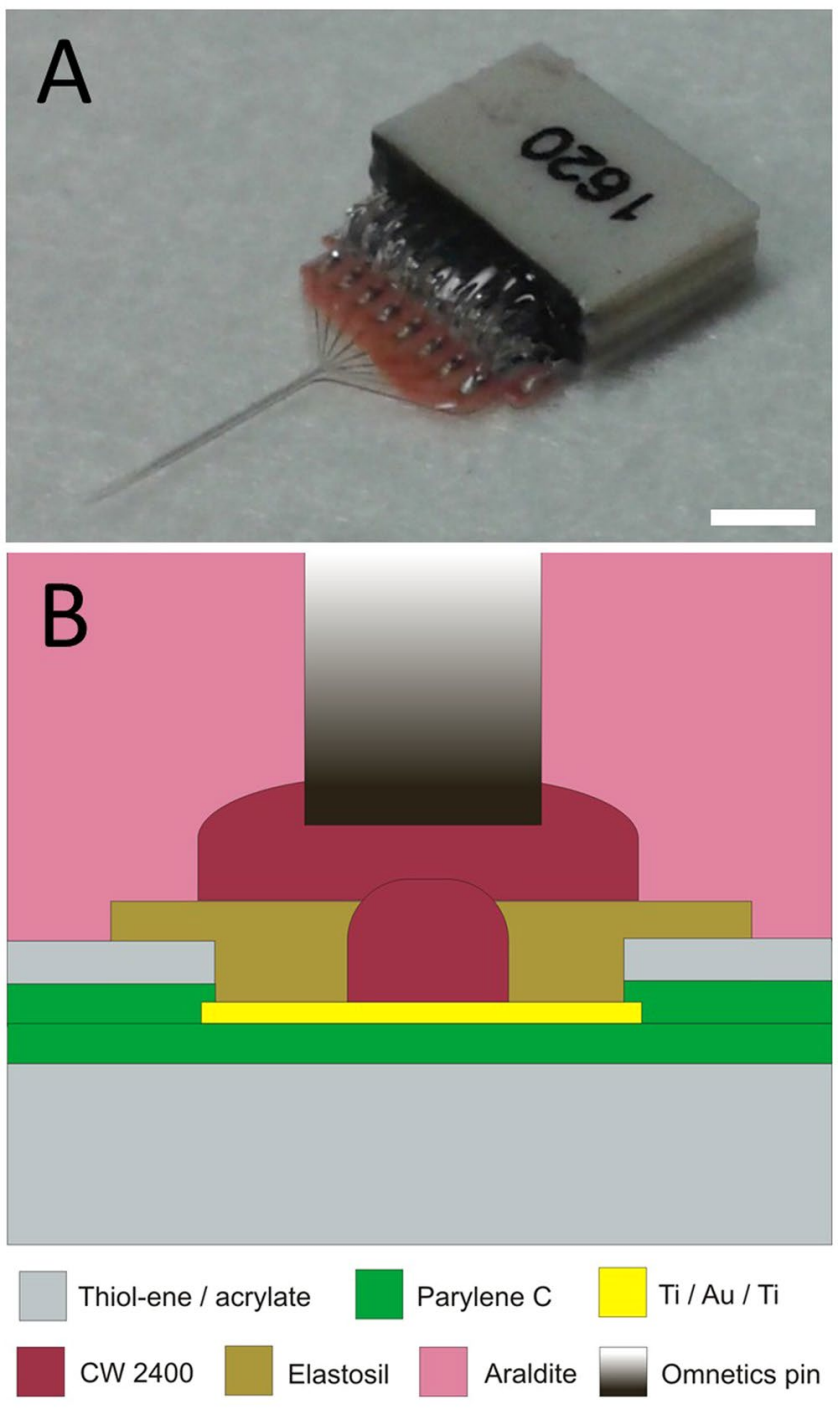
Planar microscopy view of the released probe containing gold metal traces and recording sites (**A**). Scanning electron microscopy view on the sidewall profile (**B**) on a reference recording site (**C**) and platinum coated recording site (**D**) of the released probe. High magnification images of the surface morphology of platinum black coating (**E**). Scale bars are 750 µm, 200 µm, 5 µm, 5 µm and 1 µm respectively.

The final dimension of the packaged probe is slightly bigger than the electrical connector itself, with width, length and thickness of 6.7 mm, 7.5 mm and 1.84 mm, respectively. Because reference or return electrode sites were not integrated in the probe, additional external reference and ground electrodes were used and connected to the INTAN headstage as detailed in section 2.5. The advantage of our packaging technique is that the final dimension of the probe is minimized close to the dimension of the Omnetics connector. Representative micrographs on the final device are presented in Fig. 3.

### Electrochemical characterization and electroplating

Characterization of the recording sites with electrochemical impedance spectroscopy (EIS) was performed before and after a platinum plating process and after *in vivo* use. EIS measurements were performed with a Gamry Reference 600 Potentiostat (Gamry Instruments, *Warminster*, *PA*, *US*) at 25 mV rms across a frequency range from 10 kHz to 1 Hz in phosphate buffered saline (PBS) solution (P4417, tablet diluted in 200 mL distilled water yielding 0.01 M phosphate buffer, 0.0027 M potassium chloride and 0.137 M sodium chloride, pH 7.4, at 25 °C, Merck KGaA, Germany).

In a three compartment electrochemical cell, a leakless miniature Ag/AgCl electrode (ET072-1, eDAQ Pty Ltd., Australia), a platinum wire and the softening probe sites were used as reference, counter and working electrodes, respectively. The softening probes were submerged in PBS up to bonding pads of the Omnetics connector.

Bode plots representing the impedance magnitude and phase angles of the recording sites were acquired and analyzed with Gamry Echem Analyst software (Gamry Instruments, *Warminster*, *PA*, *US*). Besides the typical measures at 1 kHz, we provide a more detailed analysis of both magnitude and phase plot throughout a broad frequency interval (10 kHz, 1 kHz, 100 Hz, 10 Hz and 1 Hz). To determine changes in the electrochemical behaviour during soaking experiments, both Bode and Nyquist impedance plots of uncoated arrays were investigated per recording sites at 37 °C.

In order to improve the active surface area, platinization of the recording sites was performed using a Gamry Reference 600 Potentiostat in galvanostatic mode. Lead free 1 wt.% chloroplatinic acid solution (diluted from 8 wt.% chloroplatinic acid solution in H_2_O, Merck KGaA, Germany) was used along with Polyvinylpyrrolidone (Polyvinylpyrrolidone, Merck KGaA, Germany) to improve the wettability of the gold surfaces. The deposition processes were carried out in a three compartment electrochemical cell. A leakless miniature Ag/AgCl (3.4 mol/L KCl) electrode (ET072-1, eDAQ Pty Ltd., Australia) and a platinum sheet were used as reference and counter electrode, respectively. Recording sites were electroplated by maintaining current density of 10 mA/cm^2^ by applying constant current around 3.14E-05 mA for cca. 60 seconds. Each site was deposited individually by adjusting the current density in order to minimize the variability of site impedances.

In order to test the electrochemical stability of the coating, four electrode sites on two individual softening probes were subjected to daily cyclic voltammetry (CV), while the other four sites on the same probes were used as references without daily CV. Stability test was carried out for nine days while constantly soaking them in 0.01 M PBS solution at 23 °C. CV curves were measured by sweeping the voltage between −0.22 to 1.0 V vs E(ref) at a scan rate of 1000 mV/sec, 20 times in the same PBS solution where the arrays were soaked. Coating integrity during 9 days of cyclic experiments were examined, experimental Nyquist plots for complex impedance were obtained via EIS. Curve fitting and equivalent circuit analysis (using Gamry Echem Analyst) were applied to the measured data employing a common method based on the Randles circuit to model the electrical characteristics of the coated surfaces. The equivalent circuit model consists of a serial resistance (R_s_) that generally describes the resistance of the bulk electrolyte combined with the internal resistance of the electrode, a charge-transfer resistance (R_CT_) that refers to the resistance of the electrode-electrolyte interface. R_L_ is the leakage resistance, the resistance associated with the pseudocapacitance in a low-frequency region^25^. CPE_L_ is related to the pseudocapacitance resulting from Faradaic processes. In parallel with R_CT_, there is a constant phase element of the double layer capacitance (CPE_DL_) featuring the interface between solid and ionic solution due to charge separation, and a Warburg element representing the diffusion of ions into the porous electrode surface.

All of the electrochemical measurements were conducted in a Faraday-cage. Dedicated arrays were used for each *in vitro* electrochemical measurements. Electrochemical impedance spectroscopy and cyclic voltammetry were carried out only *in vitro*.

### Surgery

Acute electrophysiological recordings were performed in the rat brain to test the functionality of the softening polymer based multielectrode. A total of three Wistar rats, weighing 270–400 g, were anesthetized with a ketamine-xylazine solution and prepared for stereotaxic operation as described elsewhere^26^. Animals for acute tests were kept and handled in accordance with the European Council Directive of 24 November 1986 (86/609/EEC), the Hungarian Animal Act, 1998 and the Animal Care Regulations of the Research Centre for Natural Sciences of the Hungarian Academy of Sciences (RCNS-HAS). The study was approved by the Institutional Animal Care and Use Committee of the RCNS-HAS. During anesthesia, paraffin oil was administered to their eyes to prevent them from drying. They were sacrificed by the injection of a lethal dose of ketamine/xylazine into the heart.

Craniotomy was performed −2.0 mm–6.0 mm anteroposterior (AP), 2.0 mm − 6.0 mm mediolateral (ML) in reference to the Bregma. The implantation of the multielectrode softening probes was targeted at the stereotaxic location of −4 mm AP (anteroposterior), 4 mm ML (mediolateral), perpendicularly to the brain surface, which allowed measurements in the somatosensory cortex and reaching into the hippocampus^27^.

**Figure 4:**
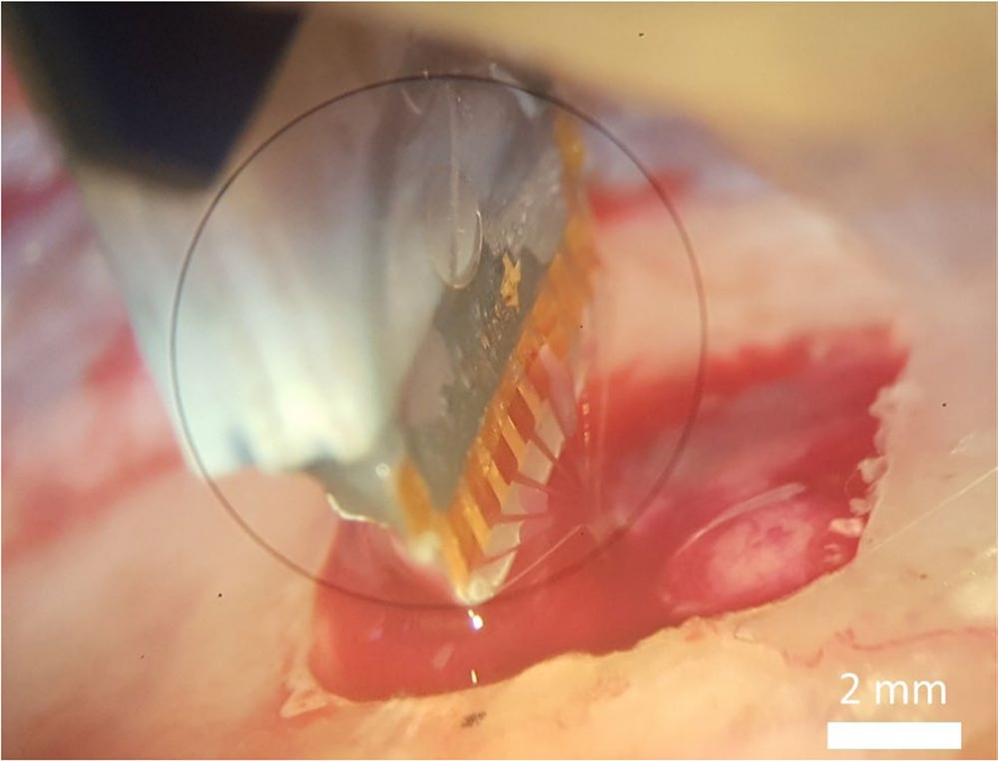
Microscopic image on the implantation of the softening probe through a skull window of a rat subject.

The dura mater was incised above the target location to achieve a smooth implantation by avoiding possible buckling of the probe. At room temperature the rigid polymer probe has proven to be suitable for inserting it into the neural tissue, without using any implantation supporting device (see Fig. 4). Insertions were made at several depths down to 4.5 mm to examine activity at different locations within the hippocampus.

### Electrophysiology and data analysis

Brain signal recordings were carried out using Intan RDH-2000 amplifier system (Intan Technologies LLC., Los Angeles, CA, USA) connected to a computer via USB 2.0, sampling with a frequency of 20 kHz. The reference electrode was a pointed stainless steel needle located beneath the skin posterior to the scalp. MATLAB 2014b (MathWorks Inc., Natick, MA, USA) was used for off-line signal visualization, filtering and analysis. In order to detect unit activity, the recordings were band-pass filtered between 500 and 5000 Hz. 50 µV was used to detect potential spikes. Principal component analysis was employed for spike sorting. The clusters were manually accepted or discarded based on spike waveforms, and they were verified by making autocorrelograms.

Single unit signal-to-noise amplitude ratio (SU SNAR) for single unit clusters was calculated as follows.

1 $$SU\,SNA{R}_{i}=\frac{P{P}_{i}}{2{\sigma }_{n}}$$

where *i* is the index of the cluster, *n* is the index of the recording channel containing the spike waveforms of cluster *i*. *PP*_*i*_ is the mean peak-to-peak amplitude of the spikes in cluster *i*, *σ*_*n*_ is the standard deviation of the filtered signal of the *n*th recording channel. Edit 4.5 software of Neuroscan (Charlotte, NC, USA) was used for analyzing the recorded low-frequency potential (LFP) signals. Epoch files were made in range of 5 ms to perform a frequency-domain data analyzis with the Fast-Fourier Transform (FFT). Calculating the power of low gamma (30–50 Hz) band of each channel evaluate the location of the electrode within the hippocampus^28^.

### Statistics

Comparison between multiple groups was performed using repeated measures ANOVA. Significant difference between the groups were found with p-value < 0.001. Post-hoc Welch’s pairwaise t-tests verified (p < 0.001) significance of the effect of soaking time, platinum deposition and *in vivo* implantation on the impedance magnitudes.

## Results

### Substrate evaluation

**Figure 5:**
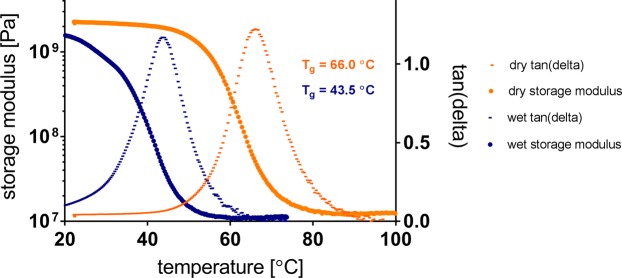
Representative dynamic mechanical analysis curves of thin film polymer samples show the difference in glass transition temperature between dry and wet conditions.

Figure 5 shows representative DMA curves of softening polymer samples with a 22.5 °C difference in glass transition temperature between dry and wet conditions, measuring 66.0 °C and 43.5 °C, respectively. Statistical analysis across fourteen batches of the same softening polymer composition have shown a standard deviation of 1.1 °C of glass transition temperature, while measurements on multiple polymer samples from the same batch have shown standard deviations of 0.1–0.3 °C of glass transition temperature. Storage modulus in the glassy state is above 2 GPa and in the rubbery state is 13 MPa (both values read from the dry measurements).

**Figure 6:**
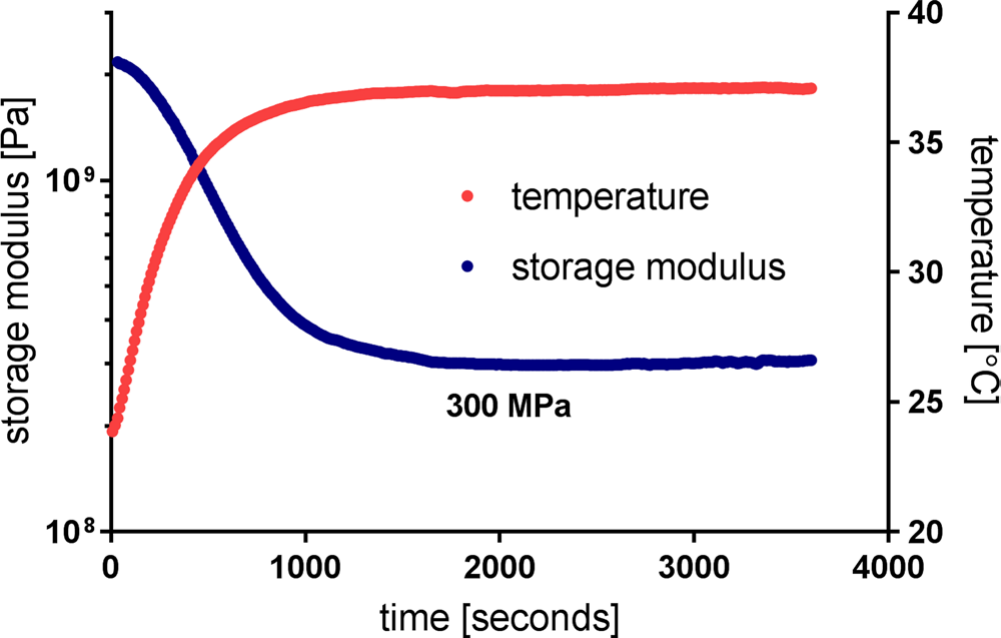
Softening of a polymer sample upon immersion into room temperature saline solution and heating to 37 °C.

The elastic modulus (storage modulus) of a softening polymer sample is tracked upon immersion in room temperature saline solution and heating from 24 °C to 37 °C, as shown in Fig. 6. The target temperature is reached in approximately 1300 seconds, while the storage modulus stabilizes at 300 MPa approximately 500 seconds later.

### *In vitro* performance

**Figure 7:**
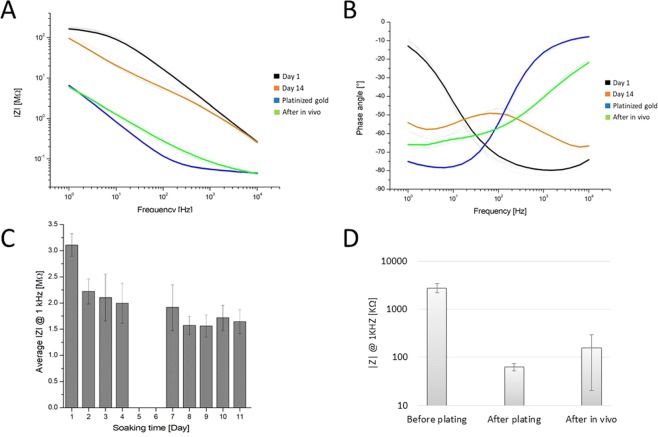
Impedance magnitude (**A**) and phase angle (**B**) of all 8 recording sites of the array. Grey lines represent single recording sites; the black, orange, blue and green lines are the average values of all sites, on the first day, the 11st day of soaking, with platinized coating and after the *in vivo* implantation respectively. Impedance magnitude measured in the soaking test at 1 kHz before electroplating is plotted against time (**C**). Change in this values throughout the lifecycle of the recording sites of two implanted probes are compared (**D**).

**Figure 8:**
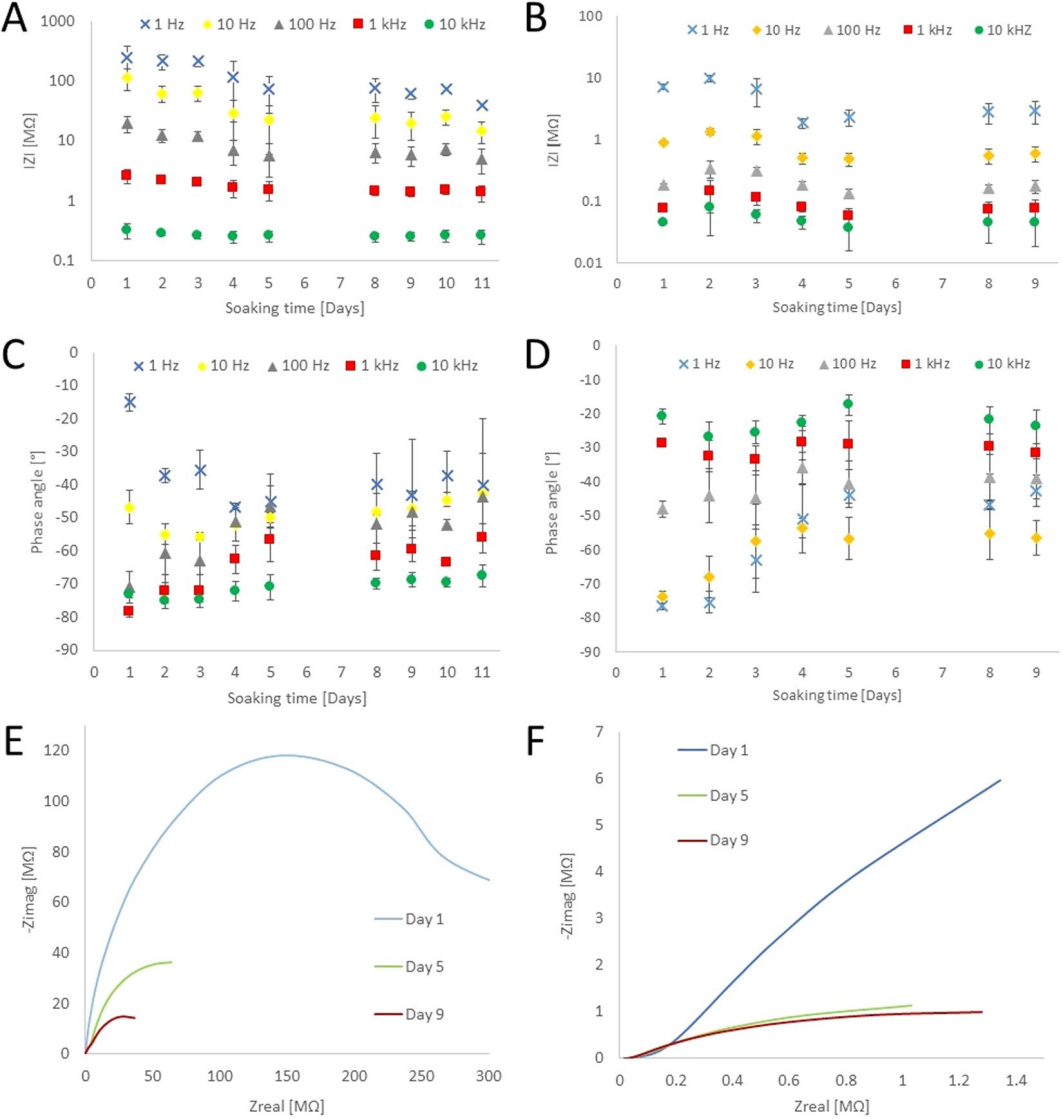
Average 10 kHz (green circle), 1 kHz (red square), 100 Hz (green triangle), 10 Hz (yellow rhombus) and 1 Hz (blue cross) impedances and phase angles of 8–8 sites on uncoated (**A**,**C**) and coated (**B**,**D**) arrays in PBS. Data present mean values ± standard deviation (n = 16). Nyquist plot is presented to show complex impedance of a representative uncoated (**E**) and coated (**F**) recording site at the 1^st^ (blue line), 5^th^ (green), 9^th^ (red) day of the soaking experiment.

The *in vitro* electrochemical measurement results are summarized in Figs 7 and 8. Before electroplating platinum on the electrode sites, the probes were soaked at 37 °C in PBS for 11 days, and EIS was recorded every day in a room temperature in 0.01 M PBS solution. Impedance magnitude and phase angle of a representative softening probe are shown versus frequency in Fig. 7A,B. Pale grey lines represent single recording sites from a 8-channel probe and the coloured lines represent the average values of all recording sites. In addition to showing Bode plots recorded on the first and the 11^th^ day of soaking, Bode plots recorded after platinum coating and after explantation from the brain are also included in this figure for comparison. Average impedance values ± standard deviation of 8 sites measured at 1 kHz before Pt deposition are shown in Fig. 7C. Since thiolene-acrylate polymers tend to slightly swell due to the water uptake (approximately 2.5 wt%), we performed the electroplating when the ultimate volume in saline is reached. After the successful soaking test, electrochemical platinum deposition of every sites were performed. Due to the electrodeposition, the impedance values of the recording sites were improved significantly by nearly two orders of magnitude from 1644 ± 160 kΩ (n = 8) down to 60 ± 11 kΩ (n = 8) (see black and blue curves on Fig. 7A,B). The measured impedance remained relatively low (83 ± 14 kΩ) (n = 8) even after the explantation of the probes after *in vivo* experiments were completed. Average impedance values of all recording sites of two implanted electrodes measured at 1 kHz are shown in Fig. 7D to illustrate impedance variation during the whole experimental procedure. Post-hoc LSD-test confirmed that improvement in impedance due to the platinum deposition and impedance change during the soaking tests were statistically significant (p < 0.001).

#### Characterization of uncoated electrode arrays

The influence of soaking on the electrochemical performance of uncoated arrays was tested at 37 °C in 0.01 M PBS solution for 11 days. EIS showed a slight decrease in the first 5–8 days of soaking in impedance magnitude between 1 Hz to 10 kHz with greater dispersion at lower frequencies (from 0.32 ± 0.09 MΩ to 0.26 ± 0.07 MΩ at 10 kHz, from 2.61 ± 0.71 MΩ to 1.36 ± 0.40 MΩ at 1 kHz, from 19.45 ± 6.10 MΩ to 5.04 ± 2.29 MΩ at 100 Hz, from 112.07 ± 43.15 MΩ to 14.74 ± 5.68 MΩ at 10 Hz, from 248.07 ± 135.98 MΩ to 40.05 ± 4.68 MΩ at 1 Hz averaged for 16 recording sites on two electrode arrays) for arrays exposed to PBS at 37 °C (Fig. 8A). After 8 days of soaking, the initial impedance values stabilized and remained stable during the 11 days of stability test. The phase angle increased at all frequencies except at 1 Hz where the greatest standard deviation (lowest precision) was observed. The greatest change in the phase angle occurred at frequencies between 100 Hz and 1 kHz (Fig. 8C).

#### Characterization of platinum-coated electrode arrays

Electrochemical performance of platinum coated arrays was tested at 23 °C in 0.01 M PBS solution. EIS showed a slight increase by the second day of soaking in impedance magnitude over the range from 1 Hz to 10 kHz (from 0.044 ± 0.002 MΩ to 0.079 ± 0.051 MΩ at 10 kHz, from 0.074 ± 0.006 MΩ to 0.142 ± 0.077 MΩ at 1 kHz, from 0.180 ± 0.010 MΩ to 0.341 ± 0.110 MΩ at 100 Hz, from 0.900 ± 0.079 MΩ to 1.330 ± 0.155 MΩ at 10 Hz, from 7.130 ± 0.714 MΩ to 9.925 ± 1.145 MΩ at 1 Hz averaged for 16 recording sites on two electrode arrays) for arrays exposed to PBS at 23 °C (Fig. 8B). Slight increase of impedance magnitude was followed by a decrease between three and four days and stabilized between four and five days over the range from 1 Hz to 10 kHz ended in a steady state after nine days of soaking (~−1.45% at 10 kHz, ~−1.96% at 1 kHz, ~3.07% at 100 Hz, ~33.07% at 10 Hz, ~58.70% at 1 Hz averaged for 16 recording sites on two electrode arrays) for arrays exposed to PBS. Increase in phase angle at lower frequencies (between 1 and 100 Hz) was observed while a slight increase was measured at 1 and 10 kHz (Fig. 8D).

Based on optical microscope investigations, the soaking process did not result in any visible delamination or degradation of the platinized electrode sites. Regarding the electrical characteristics, a significant drop in impedance magnitude and increase in phase angle were observed at lower frequencies.

After five days of soaking, impedance values stabilized around 1.36 ± 0.40 MΩ at 1 kHz for uncoated and 75.6 ± 0.03 kΩ at 1 kHz for coated sites (averaged for 16 recording sites on two electrode arrays).

#### Effects of CV on electrochemical performance

**Figure 9:**
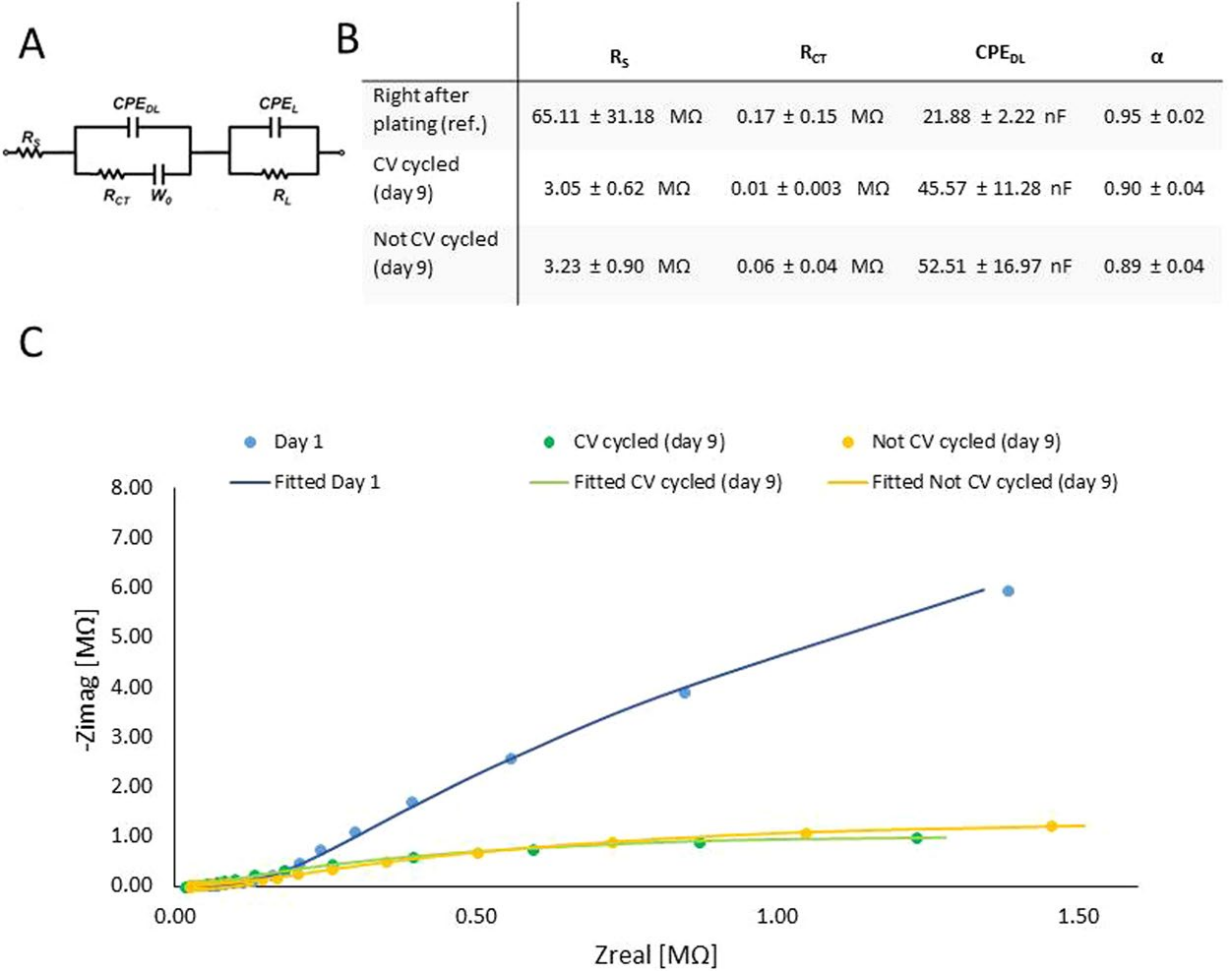
The equivalent circuit of Randle’s model for coated electrodes (**A**), where R_s_ is the serial resistance, R_CT_ in charge-transfer resistance, CPE_DL_ is the constant phase element of double layer, W_0_ is the Warburg element. Average (4–4 recording sites on 2 individual arrays) fitted model parameters right after plating, after the 1^st^ and 180^th^ cycles are shown in the inset table (**B**). Nyquist plot is presented to show complex impedance right after plating (blue circles and line) and after 9 days of soaking with (green circles and line) and without (yellow circles and line) daily CV (**C**).

Eight recording sites (four on two individual arrays) were subjected to daily cyclic voltammetry for nine days at 23 °C in 0.01 M PBS solution. The arrays were constantly soaking in the PBS solution. The equivalent circuit used for fitting of the EIS plots in this work is shown in Fig. 9A ^29^. The fitted data for relevant circuit elements is shown in the inset table of Fig. 9B. Resistance of the bulk electrolyte combined with internal resistance is represented as R_s_. As the resistance of electrolyte is constant, the decreased R_s_ after 180 cycles may correspond to electrochemical activation of active materials during cycling^29^. Charge transfer resistance (R_ct_) characterizes the rate of redox reactions at the electrode-electrolyte interface. After 180 cycles, charge transfer resistance likely decreased due to the dominant ion transport at the electrode-electrolyte interface caused by the increased contact area. Constant phase element (CPE) represents double layer capacitance, which occurs at interface between solids and ionic solutions due to separation of ionic and/or electronic charges^30^.

The CPE is modeled using two terms: Y, the coefficient of CPE capacitance per unit length and α, a parameter defined by the phase angle of the CPE. α has values between 0 and 1, where α = 1 represents an ideal capacitor with phase angle 90° and is proportional to surface roughness, charge uniformity etc^25^. Based on our fitted parameters, α slightly changed in both cases (CV and non CV stimulated sites). Since the diffusion path length of the ions in the electrolyte in our test conditions is short, value of Warburg impedance (W_0_) remained in the same order of magnitude during the experiment (from 8.45*10^−8^ − 5.33*10^−7^ to 3.44*10^−7^ and 3.53*10^−7^ in CV and non CV cases, respectively). Leakage resistance (R_l_) is usually very high and can be ignored in the circuit. No significant change in pseudocapacitance (CPE_l_) is observed. The Nyquist plots are fitted according to the model, and the as-fitted Nyquist plots on the 1^st^ and the 9^th^ day are shown in Fig. 9C.

### *In vivo* performance

The softening polymer based neural probes proved to be suitable for precise insertion into the neural tissue and capable of detecting spikes from the hippocampus. Implantation was successful and the probes were inserted without any problem of bending or buckling in all three test animals. The maximum observed peak-to-peak amplitude was 84 μV with the signal-to-noise (SNR) ratio of 6.24, whereas the maximum number of units identified from the analysis was 4 per probe.

**Figure 10:**
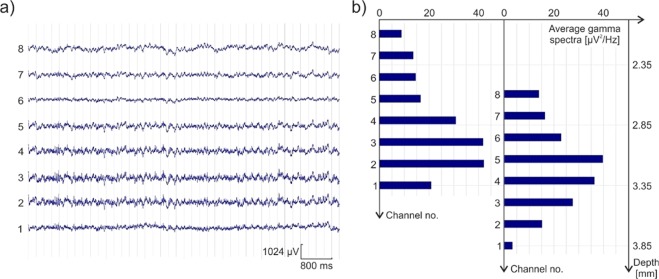
Representative sample of recorded LFP signals (**a**), and the gamma power of channels according to their depth in the brain (**b**).

A representative sample of the recorded LFP signals is shown Fig. 10a. In Fig. 10b the associated power in the gamma band per channel is illustrated. This power correlates with the proximity of each electrode with the hippocampus. The left part of Fig. 10b shows a case when channel #2 and #3 were located in this region. This measurement was followed by advancing the probe 500 µm deeper into the tissue, which allowed channel #5 to collect the highest amplitude gamma waves, as expected in view of the spacing of recording sites.

**Figure 11:**
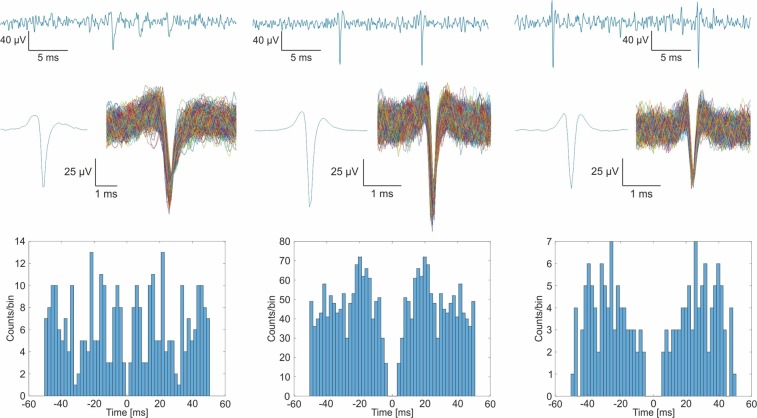
Representative filtered waveform of the acute test, samples of the clustered spikes from each implantation and their autocorrelograms.

Representative sample of the 500–5000 Hz band-pass filtered waveforms is presented from each acute tests in Fig. 11.

Detected spikes can be observed along the waveforms. The result of the spike sorting are presented under the filtered waveforms: the average of one clustered spike from each waveform (on the left), and all their occurrences. The autocorrelograms of the presented spikes are shown in the lower part of Fig. 11. The autocorrelogram specifies the probability of encountering the same spike as a function of time after a given spike. Refractory periods are observable in the middle of the autocorrelograms and indicate correctly identified clusters.

## Discussion

Prior to the present study, the use of softening polymers in the investigations of the central nervous system was so far limited to intracortical recordings. Various approaches to aid the device insertion of thin compliant neural interfaces into the cortex include the use of shuttles^31^ or dissolvable coatings^32,33^. In order to go beyond cortical layers, we employed a substrate thickness of 60 micron, which provided proper guidance towards the targeted brain region. Soft state elasticity was limited to 300 MPa in our study. While softening polymer compositions that provide a lower elastic modulus upon implantation are available^34^, we chose this composition in anticipation of better handling during our acute study that used probe insertion and positioning times extending beyond the transition time of the polymer. Our *in vitro* studies suggest that our fabrication and packaging technology provides a stable softening neural interface with very small device footprint.

Thiol-ene/acrylate shape memory polymer compositions have been shown to soften from over 1 GPa to 18 MPa with less than 3% fluid uptake upon exposure to 37 °C PBS^18^. The DMA results presented in this work predict a 300 MPa storage modulus upon implantation, an order of magnitude higher than the 23 MPa storage modulus reported for the same composition after one week of implantation by Ware *et al*.^18^. The main reason for this difference is in the polymerization process. Ware *et al*. used 15 minutes curing with exposure to 365 nm UV light through a glass mold; while in this work, open surface spin-coated monomer solution has been polymerized by a combination of 30 seconds exposure to 254 nm UV light, 60 minutes exposure to 365 nm UV light and a 24 hours long post-curing session in vacuum oven. The extended curing process ensures that polymerization progresses to completion and the cross-linking is maximized. As a result of the increased cross-link density in the polymer, the dry Tg is shifted from 58 °C to 66 °C, and the material no longer softens to its potential 13 MPa rubbery modulus (see in Fig. 5), but instead exhibits 300 MPa modulus in the simulated physiological environment (see in Fig. 6). While the Tg shift is not necessarily a desired outcome, the extended curing process was a necessary change in order to stabilize the polymer substrate for thin film microfabrication processes such as plasma enhanced chemical vapor deposition. Considering that the relationship between intracortical probe stiffness alone and the foreign body response that follows the traumatic injury of implantation is not well understood, we argue that the technology presented here provides a valuable tool for research.

Results of impedance and phase angle analysis during soaking period (11 days for uncoated, 9 days for coated) in PBS solution revealed a decrease in impedance and an increase in phase angle that are likely due to diffusion of water and solutes into the polymer layers^35^. Uniform decrease in the impedance suggests a possible penetration of moisture through the polymer layers, resulting in a switch from capacitive to dominantly resistive state^36^. Almost uniform increase in the phase angle at all frequencies supports the hypothesis that the resistive mode of conductivity dominates^37^. Due to the Parlyene capping of the electrical traces, these phenomena do not deteriorate device performance after an equilibrium of impedance values have been reached.

Electroplating platinum black on the electrode sites lowers their impedance to approximately 1/50 of that of the original gold site, granting them to be the primary pathway for current flow. In our study, we examined EIS data at five different frequencies in order to more accurately predict the failure mechanism of insulation layers^37^. Nyquist plots with greater linear behavior revealed that the impedance decrease after plating is due to the improvement of surface capacitance by an order of magnitude, which is likely caused by the porous structure of the coating. We also found that the porous platinum layer was not damaged during the insertion procedure (see blue and green lines on Fig. 7A,B). Optical microscopic observation did not show any visual change in appearance due to possible delamination. Changes in fitted parameters confirmed the electrochemical stability of platinum black coatings. It is notable that R_s_ values also reduced when recording sites were not subjected to daily CV, consequently the change of this parameter may refer to the diffusion of ions through the polymer during soaking in PBS. To conclude the results of CV cycling, this test had only a slight effect on the performance, while soaking conditions had a more dominant influence on the variation of fitted parameters.

During *in vivo* validation of the proposed device, we presented un-aided acute implantation (without the use of an insertion shuttle) of a 5 mm long, 60 um thick and 200 um wide softening laminar brain probe in the rat hippocampus and demonstrate neural recordings from 3.85 mm depth. Neuronal spiking activity was detected during our *in vivo* study with a maximum SNR of 6.24. This SNR is somewhat above the value reported by Ware *et al*.^18^ using a softening probe of electroplated recording sites. Reaching the hippocampus, not only provides access to the center of memory and spatial navigation with shape-memory polymer based probes, but opens up new opportunities to conduct experiments in even deeper areas of high interests for the field of neurodegenerative research. In our study, the softening probes were easy to handle and all implants maintained their shape without fracturing or bending. Our experiments imply that smart implants made of mechanically adaptive polymers are promising candidates to replace rigid laminar microelectrodes used for monitoring and stimulation of the deep neural tissue.

## Conclusion

In our work, a softening polymer neural probe was fabricated and implanted into a rat hippocampus to record the firing activity of neurons. The probe changes its elastic modulus from 2 GPa to 300 MPa when exposed to physiological conditions in a 10 minute timescale. The 5 mm long, single shank, multi-channel probes are composed of a custom thiol-ene/acrylate thermoset polymer substrate, and were micromachined by standard MEMS processes. Electrochemical properties of the probe were stable under soak testing *in vitro*, and were retained after the implantation process. Single unit activity was recorded in acute experiments with typical spike amplitudes of 84 µV and signal-to-noise ratio of 6.24. In the future, chronic *in vivo* investigations will be made to demonstrate long term neural recording performance from the rat hippocampus, and to compare with that of silicon probes of the same dimensions in order to investigate the influence of the as-reduced mechanical mismatch between our device and the brain tissue on the neuroinflammatory response and device functionality.

## Data Availability

The datasets generated during and/or analysed during the current study are available from the corresponding author on reasonable request.
